# Therapeutic Strategies for Retinal Artery Occlusion—A Literature Review

**DOI:** 10.3390/jcm13226813

**Published:** 2024-11-13

**Authors:** Joanna Roskal-Wałek, Alicja Ruzik, Natalia Kubiś, Maria Teper, Michał Wesołowski, Zuzanna Wujec, Paweł Wałek, Dominik Odrobina, Jerzy Mackiewicz, Beata Wożakowska-Kapłon

**Affiliations:** 1 Ophthalmology Clinic, Voivodeship Regional Hospital, 25-736 Kielce, Poland; 2 Collegium Medicum, Jan Kochanowski University, 25-516 Kielce, Poland; alicjaruzik@gmail.com (A.R.); natalia.kubis.nk@gmail.com (N.K.); teper.m@interia.pl (M.T.); michal_wesolowski1@wp.pl (M.W.); zuzannna.wu@gmail.com (Z.W.); pwalek@ujk.edu.pl (P.W.); magdale_5@hotmail.com (D.O.); bw.kaplon@poczta.onet.pl (B.W.-K.); 3 Department of Pathophysiology, Wroclaw Medical University, 50-368 Wrocław, Poland; 4 1st Clinic of Cardiology and Electrotherapy, Swietokrzyskie Cardiology Centre, 25-736 Kielce, Poland; 5 Ophthalmology Clinic Boni Fratres Lodziensis, 93-357 Łódź, Poland; 6 Department of Vitreoretinal Surgery, Medical University of Lublin, 20-079 Lublin, Poland; jerzy.mackiewicz@umlub.pl

**Keywords:** retinal artery occlusion, stroke, retina, hyperbaric oxygen therapy, thrombolysis

## Abstract

Retinal artery occlusion (RAO) is an emergency condition causing acute retinal ischemia and is considered an equivalent of ischemic stroke. The occurrence of an episode of RAO is associated with significant impairment of visual functions and correlates with an increased risk of future vascular events. Although RAO requires immediate diagnosis and treatment, there are currently no clear guidelines specifying optimal management. This review discusses current and future therapeutic strategies following an episode of RAO, including secondary prevention.

## 1. Introduction

Retinal artery occlusion (RAO) is one of the causes of acute retinal ischemia. There are two forms of RAO: central retinal artery occlusion (CRAO) and branch retinal artery occlusion (BRAO) [1,2]. CRAO has an incidence of 1.8/100,000 person-years, but this incidence rises to 10/100,000 in those over the age of 80. It is assumed that the episodes of BRAO constitute 38% of all RAO cases [3,4,5].

Most CRAO cases are associated with a significant decrease in visual acuity (VA), while BRAO causes less severe visual loss with local visual field defects that may be unnoticed by the patient [1,2,5]. RAO can be divided into non-arteritic RAO (NA-RAO), which accounts for the majority of cases, and arteritic RAO (A-RAO), which is most often associated with giant cell arteritis (GCA) [1,2,5]. It is believed that NA-RAO is most often caused by embolic material originating from the ipsilateral carotid artery, heart, or aortic arch [1,2,5,6,7]. Recently, it has been shown that inherited and acquired thrombophilic risk factors play an important role in the pathogenesis of CRAO [8]. Studies have demonstrated that in cases of RAO resulting in ischemia lasting more than 240 min, irreversible ischaemic lesions occur in the retina [9]. Therefore, in RAO, the time to implement appropriate therapeutic intervention is crucial. In addition to the risk of irreversible vision loss, RAO patients are also at an increased risk of subsequent vascular events and higher mortality compared to control groups, which applies to both CRAO and BRAO patients [5,10,11]. The American Heart Association (AHA) and the American Stroke Association (ASA) recognized acute retinal ischemia as the equivalent of acute cerebral ischemia [12]. The current preferred practice patterns proposed by the American Academy of Ophthalmology (AAO) recommend urgent diagnostics in RAO patients, analogous to that dedicated to patients with ischemic stroke, to reduce the risk of subsequent stroke and vascular events [2,13]. The recognition of RAO as a stroke once again directs our attention to the currently used treatment methods, and in particular, to the recommendations regarding the use of thrombolysis [14,15,16,17,18,19,20,21,22,23,24,25], as well as to the recommendations for secondary prevention in this group of patients [15,17,26,27,28].

The range of therapeutic methods used in RAO has expanded over the years [6,7,8,14,15,16,17,18,19,20,21,22,23,24,25,29,30,31,32,33,34,35,36,37,38,39,40,41,42,43,44,45,46,47,48,49,50,51,52,53,54,55,56,57,58,59,60,61,62,63,64,65,66]. However, despite many years of clinical research, no consensus has been reached on the most effective method of treating RAO. There are no clear, commonly accepted guidelines indicating the therapeutic algorithm in the case of RAO. The currently applied methods include conservative treatment [6,7,17,18,33,34,35,36,37,38,39,40,41,42,43,44,45,46,47,48,49,50,51,52], thrombolysis [14,15,16,17,18,19,20,21,22,23,24,25], transluminal Nd:YAG laser embolysis (TYL) [53,54,55,56,57,58,59], and vitrectomy [60,61,62,63,64,65]. Research on the use of Kyoto University Substance (KUS121) is underway [66]. The efficacy of these treatments is controversial, and many of them have ambiguous risk profiles, which also translates into differences in the selection of specific treatments used in clinical practice (Table 1).

Over the years, there has been a marked improvement in the quality of the studies conducted. Through case series, individual original studies based on very small groups, several randomized controlled trials, and meta-analyses that considered selected treatment methods, we are now awaiting publication of the results of ongoing large clinical trials [6,7,8,14,15,16,17,18,19,20,21,22,23,24,25,29,30,31,32,33,34,35,36,37,38,39,40,41,42,43,44,45,46,47,48,49,50,51,52,53,54,55,56,57,58,59,60,61,62,63,64,65,66]. Because of the heterogeneity of the studies, it is not possible to conduct meta-analyses comparing all available treatment methods. The authors of a Cochrane systemic review, due to the heterogeneity of the studies, were unable to conduct any meta-analyses. They ultimately evaluated six randomized controlled trials, comparing any interventions with another treatment in patients with acute non-arteritic CRAO. The authors concluded that the proposed interventions for acute noninflammatory CRAO may not be better than observation or treatment of any kind, such as eye massage, oxygen inhalation, tube dilation, or anticoagulation, although the evidence is uncertain [30].

In this work, we present a review of the literature on treatment methods used in the case of RAO, including a discussion on secondary prevention that should be implemented, considering that RAO is a stroke equivalent.

## 2. Materials and Methods

In June 2024, an extensive manual search was conducted through the major electronic databases (PubMed) in order to identify relevant studies focused on the treatment and secondary prevention of RAO. In the search criteria, original articles, literature reviews, and case studies from 1980 to 2024 were marked due to the importance of the issues. The following search terms were used: ‘retinal artery occlusion treatment’, ‘retinal artery occlusion secondary prevention’, ‘retinal artery occlusion conservative treatment’, ‘retinal artery occlusion hyperbaric oxygen therapy’, ‘retinal artery occlusion thrombolysis’, ‘retinal artery occlusion transluminal Nd:YAG laser embolysis/embolectomy’, ‘retinal artery occlusion vitrectomy’, and ‘KUS121′ in different combinations. A total of 83 compatible research publications were identified and used to compile this review.

## 3. Discussion

### 3.1. Conservative Treatment of Retinal Artery Occlusion

Many conservative methods have been used to restore blood flow to the retina and improve visual function in RAO cases. Possible treatments include pentoxifylline (PXF), paper bag breathing, carbogen therapy (95% O_2_/5% CO_2_), sublingual isosorbide dinitrate, ocular massage, anterior chamber paracentesis, intraocular pressure-lowering agents, acetazolamide, mannitol, methylprednisolone, and hyperbaric oxygen therapy (HBOT) [5,6,7,17,18,33,34,35,36,37,38,39,40,41,42,43,44,45,46,47,48,49,50,51,52]. The common goal of all treatment methods is to improve or restore retinal circulation before retinal necrosis occurs, using a variety of potential mechanisms [5,47].

#### 3.1.1. Mechanism: Increase in Erythrocyte Deformability

##### Pentoxifylline

PXF reduces the stiffness of erythrocytes, facilitating their passage through capillaries, thus increasing blood flow and tissue perfusion and reducing ischemia [7].

In the study by Incandela et al., the authors showed that the use of 1800 mg PXF (3 × 600 mg) for 4 weeks in patients with CRAO increased peak-systolic flow (PSF) by up to 550% in the treated group vs. 288% in the placebo group and increased end-diastolic flow (EDF) by up to 400% in the PXF group vs. 200% in the placebo group. PXF is generally safe and well tolerated [47]. Moreover, as shown in the study by Iwafune et al., the use of PXF prevents the development of neovascularisation caused by retinal ischemia [34]. However, due to the lack of evidence to support PXF’s ability to affect visual outcomes, routine administration of this drug in CRAO patients is unsubstantiated [47]. PXF cannot be used by people with an allergy to theophylline or caffeine. Adverse effects of this treatment include dehydration, constipation, anorexia, cholecystitis, aseptic meningitis, seizures, confusion, depressive symptoms, hypotension, edema, dyspnoea, epistaxis, respiratory distress, dermatitis, angioedema, urticaria, pruritus, otalgia, scotomas, conjunctivitis, and blurred vision [7].

#### 3.1.2. Mechanism: Vasodilation

##### Rebreathing into a Bag

The mechanism of CRAO treatment by breathing into a paper bag involves inducing hypercapnia, which increases blood flow in the retinal vessels, and hypoxia, which causes vasodilation of the vessels. Moreover, hypercapnia and hypoxia combine to increase cardiac output and arterial blood pressure, leading to higher ocular perfusion pressure values. These mechanisms are meant to move the embolus [35,36].

However, inducing hypercapnia and hypoxia is associated with certain systemic risks, as well as the risk of hypoxia in the already ischaemic retina. Nevertheless, if this method is used for short, 1–2 min periods, the short-term hypoxia is of little consequence compared to the benefits of potential embolus displacement [35].

This method has certain limitations. It can only be used in young people with no history of cardiovascular conditions. This is problematic because most patients with CRAO are elderly people suffering from atherosclerosis, arterial hypertension, and other cardiovascular diseases [35].

##### Inhalation of Carbogen

Carbogen is a mixture of gases containing 5–6% carbon dioxide and 94–95% oxygen [36,37,38,39]. The theoretical mechanism of action of this mixture is to increase retinal oxygenation through high oxygen concentration. However, oxygen itself causes retinal vasoconstriction, so it was assumed that adding carbon dioxide, which has an antagonistic vasodilatory effect, would counterbalance the vasoconstrictor effect of oxygen and perhaps even cause mild vasodilation. In the study by Atebara et al., patients with acute NA-CRAO were treated with anterior chamber paracentesis and carbogen therapy. Despite the use of both methods, the benefits of treatment were insignificant [37].

In the study by Arend et al., no change in the diameter of the arteries was demonstrated after the application of this method. However, a significant increase was observed in the vascular flow rate and arteriovenous passage time, which may improve oxygenation [38].

##### Isosorbide Dinitrate = Nitroglycerine

Isosorbide dinitrate causes vasodilation, vascular resistance reduction, and blood flow improvement. In the context of CRAO treatment, nitroglycerine was studied only as a part of combined treatment [39]. For this reason, it is not possible to directly assess the effect of nitroglycerine on the course of CRAO [5,7,17,39]. Contraindications to the use of this treatment method include hypersensitivity, severe anemia, and recent use of phosphodiesterase inhibitors [7].

Potential risks associated with the above-mentioned methods connected with the vasodilation mechanism include the dilation of blood vessels in the circulatory system, which causes a decrease in systemic blood pressure [17].

#### 3.1.3. Mechanism: Increase of Partial Pressure of Oxygen in the Blood

##### Hyperbaric Oxygen Therapy

Hyperbaric oxygen therapy (HBOT) involves breathing in almost 100% oxygen in a hyperbaric chamber, where the pressure is higher than the pressure at sea level, which increases the concentration of oxygen in the blood and allows it to diffuse to the retina through the choroidal circulation [40].

Under normal conditions, the retina receives most of its oxygen via central retinal artery (CRA) perfusion, but this function is also partially performed by the choroidal vessels. In hyperbaric oxygen therapy, the increased oxygen content in the respiratory mixture (FiO_2_), supplied by means of diffusion from the choroid and possible residual retinal arterial perfusion, allows retinal cells to survive until vessel recanalization [6,40]. The study by Li et al. demonstrated that hyperoxia may meet the 100% oxygen demand of the retina [41].

Typically, the therapy uses a pressure of 2 absolute atmospheres (ATA), which may be increased up to 2.8 ATA depending on the results. One cycle lasts about 90 min. The most efficient total treatment duration is over 9 h [42].

Many studies have confirmed the efficacy of HBOT in improving VA in CRAO patients. Most of these studies were conducted in patients who presented no later than 24 h after the onset of symptoms [43,44,45,46]. However, a recent meta-analysis showed that HBOT does not improve final visual outcomes, visual benefits might exist for a select number of patients that receive treatment shortly after the onset of RAO symptoms [40].

Moreover, it is necessary to consider a serious complication of HBOT, i.e., barotrauma, ear pain, ruptured eardrum, and generalized seizures [40]. It was also assumed that HBOT therapy itself may contribute to the development of ocular neovascularization after CRAO [6].

#### 3.1.4. Mechanism: Intraocular Pressure Reduction Leading to Retinal Perfusion Pressure Increase

##### Eye Massage

The eye massage method is performed in two ways. The eye is gently massaged through the closed eyelid by a physician applying pressure using fingers or a Goldmann three-mirror lens placed directly on the eye-globe [7,17,36,46,47].

There are no detailed guidelines regarding the time of applying the pressure. One of the techniques involves continuous eye massage for 15–20 min [48]. It is also possible to use the intermittent method—eye massage with the application of increased pressure for 10–15 s, followed by an abrupt release of the pressure for 3–5 min [18,47], as well as mixed methods [39].

When the pressure is released, there is a sudden decrease in intraocular pressure (IOP) and, consequently, an increase in retinal perfusion pressure, which, in theory, should facilitate the disintegration and displacement of the embolus to the peripheral vessels [36,47,48].

This method is widely used due to its simplicity and lack of a need for specialized equipment, but there is no research on its standalone effectiveness. It is usually used in combination with other methods, the outcomes of which have not demonstrated any significant benefits [48].

##### Acetazolamide

Acetazolamide is a carbonic anhydrase inhibitor that reduces the production of aqueous humor and thus reduces IOP, which leads to increased retinal vascular perfusion. Acetazolamide can be administered intravenously (500 mg) or orally in a short-term therapy, and, in combination with eye massage, it is probably the most common treatment for CRAO [48].

The effect of acetazolamide was assessed by Rassam et al. The authors observed increased retinal flow after intravenous administration of 500 mg of acetazolamide in 10 healthy patients [49]. However, in the case of CRAO, acetazolamide is usually used in combined therapy, which makes it difficult to clearly assess its effectiveness [18,39].

The use of acetazolamide, especially in prolonged therapy, is associated with a large number of adverse effects, such as paraesthesia, a metallic/bitter taste in the mouth, and fatigue. In rare cases, the use of acetazolamide may lead to life-threatening anaphylaxis, metabolic acidosis, Stevens–Johnson syndrome, and blood dyscrasias [47].

Other IOP-lowering drugs used in CRAO include glycerol and mannitol [39,47].

##### Anterior Chamber Paracentesis

Anterior chamber paracentesis involves inserting a 27–30 (gauge) needle into the anterior chamber and then withdrawing 0.1–0.4 mL of aqueous humor, which results in a significant IOP decrease and widening of the retinal vessels, allowing the embolus to move to smaller arterioles that are not as important for central vision [37,48].

Fieß et al. compared 15 CRAO patients receiving conservative treatment to a group of 59 patients subjected to conservative treatment combined with anterior chamber paracentesis. There was no additional gain in visual acuity after paracentesis, regardless of the time elapsed between the first symptoms of CRAO and the performance of paracentesis. Moreover, the paracentesis procedure may be associated with the occurrence of complications [50]. The most common ones include inadvertent ocular trauma, intraocular hypotony, corneal decompensation due to iridocorneal touch, intraocular hemorrhage, and intraocular infection [17,50]. In the study by Fieß et al., one patient suffered a lens injury, with a subsequent need for cataract surgery [49]. Another limitation is the need to repeat the procedure, as the anterior chamber fills up again within 1–2 h after the procedure [46,48].

##### Topical Medications for IOP Lowering

Their mechanism of action involves a reduction in aqueous humor production by beta-adrenergic blockers or carbonic anhydrase inhibitors or an increase in uveoscleral outflow by prostaglandin analogs. Alpha2-adrenoceptor agonist acts by reducing aqueous humor production and increasing uveoscleral outflow. Despite their effectiveness, the onset of action is slow compared to acetazolamide and mannitol [7].

#### 3.1.5. Mechanism: Reduction of Retinal Edema

##### Methylprednisolone

If CRAO is caused by vascular endothelial edema, rapid recanalization is possible with steroid therapy [51,52].

In a single case series study of 4 patients with acute CRAO, Hausmann et al. administered 1000 mg of methylprednisolone in an intravenous bolus to patients who had not responded to conventional therapy (acetazolamide and anterior chamber paracentesis). As a result, 3 out of 4 patients regained efficient retinal circulation, with almost normal circulation times 60 min after methylprednisolone administration. Atherosclerotic lesions were probably responsible for thrombosis in the patient whose treatment was ineffective. Methylprednisolone acts effectively in patients with vasospasm or CRAO secondary to vasculitis by relieving endothelial edema. The authors emphasized that methylprednisolone did not interact with other drugs used to treat CRAO [51]. Other researchers, however, indicated adverse effects that, although rare, can be serious, including anaphylactic reactions, tetraplegia, cardiac arrhythmias, and sudden death [52]. According to a meta-analysis conducted by Schrag et al., patients who received an ocular massage, anterior chamber paracentesis, and/or hemodilution had a visual recovery rate of 7.4% compared to 17.7% in the natural history. Moreover, the analysis suggests that conservative treatments are futile and may be harmful [16]. The current American Academy of Ophthalmology (AAO) Practice Pattern does not recommend conservative therapy [2].

### 3.2. Thrombolysis

The current AHA guidelines for the treatment of acute ischaemic stroke do not include CRAO [67]. However, since CRAO is considered an ocular analog of ischaemic stroke, it may be beneficial to use thrombolytic therapy to dissolve the clot blocking the artery in the same manner as in ischaemic stroke treatment [14,15,17,18]. The literature data indicate that most emboli in CRAO are composed of cholesterol or calcium; therefore, the effectiveness of thrombolysis may seem questionable. However, in the case of all major causes of CRAO—carotid artery disease, valvular disease, atrial fibrillation (AF), and thrombophilia—the emboli are fibrin-related, even if they contain calcium or cholesterol crystals. Furthermore, embolic material captured during carotid artery stenting and transcatheter aortic valve replacement usually consists, at least partly, of thrombus. This suggests that even if the vessel-blocking molecule contains crystalline elements, it may still be subject to thrombolysis [8,24].

Attempts at such treatment have been made since the 1960s. Thrombolytic treatment is used in 5.8% of CRAO patients in the US [68]. The medications used in thrombolytic therapy include alteplase, i.e., tissue plasminogen activator (tPA), and tenecteplase (genetically modified tPA, which can also be administered in bolus) [14]. Currently, only alteplase is used in the treatment of CRAO [14], but research on the use of tenecteplase is ongoing; for example, the Tenecteplase in Central Retinal Artery Occlusion Study (Ten-CRAOS); ClinicalTrials.gov ID: NCT04 526 951) [28].

In the case of RAO, intravenous thrombolysis (IVT) and intra-arterial thrombolysis (IAT) were used. Due to uncertain results regarding the effect of these methods on the final visual acuity, as well as the risk of adverse events, to date, they have not been approved as a standard of care [29].

In a recent metanalysis, Liu et al. showed that both IVT and IAT are effective in treating CRAO. Compared to the standard treatment, thrombolytic strategies were associated with a higher risk of adverse events, but these strategies did not differ. Based on the results of the subgroup analysis, the authors speculate that IV tPA injection within 4.5 h of the onset of CRAO should be the optimal thrombolytic option for treating CRAO [29].

#### 3.2.1. Treatment Protocol for Central Retinal Artery Occlusion

The procedure of initiating thrombolytic therapy in a CRAO patient begins with an urgent ophthalmologic examination performed concurrently with a neurological assessment using the National Institutes of Health Stroke Scale (NIHSS) (Table 2) and a non-contrast computed tomography (CT) scan of the head. If coagulation disorders are suspected, the platelet count, international normalized ratio (INR), and activated partial thromboplastin clotting time (aPTT) should be determined. If inflammatory causes of CRAO, such as GCA, are suspected, the erythrocyte sedimentation rate (ESR) and C-reactive protein (CRP) must be determined. Ultrasound of temporal and/or axillary arteries is recommended as the first imaging modality in patients with suspected GCA. A halo sound is the ultrasound finding most suggestive of GCA [31]. In the case of arteritic CRAO, high doses of intravenous methylprednisolone should be used, and a temporal artery biopsy should be performed. In the case of non-arteritic CRAO, IVT should be considered in the absence of contraindications within 4.5 h of symptom onset. In case the patient is not a candidate for IVT and 6 h have not passed, IAT tPA may be considered if available [15].

#### 3.2.2. Intravenous Thrombolysis with tPA

IVT is applied in the same manner as in ischaemic stroke treatment. This means that tPA is administered intravenously at a dose of 0.9 mg/kg (the maximum dose of 90 mg should not be exceeded). Ten percent of the total dose is administered as an initial intravenous bolus over 1 min, with the remainder of the dose administered in a 1-h infusion [14]. This treatment is most effective if administered within 4.5 h from the occurrence of the first symptoms [15].

Before the application of IVT, it is imperative to exclude intracranial or other significant hemorrhages and to exclude the presence of ocular contraindications, which include increased IOP above 30 mmHg, VA better than 0.1, clinical or laboratory evidence of temporal arteritis, combined arteriovenous occlusion, BRAO, CRAO without foveal ischemia, active choroidal neovascularisation, proliferative diabetic retinopathy, and other severe retinopathy. Contraindications to the use of IVT also include uncontrolled blood pressure, effective anticoagulation treatment, hemorrhagic diathesis, significant aneurysm or arterial dissection, as well as infective endocarditis and peripartum period [14].

In 2015, Schrag et al. conducted a meta-analysis demonstrating that clinical improvement was achieved in 50% of CRAO patients who received any lytic drug within 4.5 h from the onset of symptoms. The CRAO patients were divided into three groups. A total of 396 patients received no treatment, 419 patients were treated conservatively, and 147 patients were subjected to IVT using various fibrinolytic agents. In the group of 34 patients who received IVT within 4.5 h of the onset of symptoms, 17 (50%) achieved visual function recovery, whereas no effects were observed in patients treated after the 4.5 h mark. The recovery of visual function was defined as a final VA score of 20/100 or better in the affected eye when the baseline uncorrected VA was 20/200 or worse [16].

Following the publication of the meta-analysis results, Mac Grory et al. conducted a study evaluating tPA treatment administered to CRAO patients within 4.5 h after the onset of symptoms. The updated analysis also demonstrated a considerable effect of treatment implemented within the 4.5-h period [19].

Janská et al. analyzed the results of 6 studies that addressed the efficacy of RAO treatment using IVT with tPA. Of the total sample of 58 patients who received tPA within 4.5 h from the onset of symptoms, VA improved in 26 (45%). In 25 patients who received treatment 4.5–6 h after the onset of symptoms, the effect of IVT was observed in 13 (52%). When IVT was administered over 6 h after the onset of symptoms, the effect of IVT was confirmed in just one patient [14].

The only randomized, double-blind study to date was published by Chen et al. in 2011. It included 16 patients with symptoms lasting for 4 to 24 h. Eight patients received IVT, but only one of them received thrombolytic treatment within 4.5 h, and one within 6 h after the onset of symptoms. These patients showed significant improvement in VA by more than three lines on the Snellen chart at 1-week follow-up. However, the improvement in VA was not sustained, and a deterioration in VA was observed at 6-month follow-up. An adverse event that occurred in one patient in this study was symptomatic intracranial hemorrhage; however, this patient was diagnosed with cerebral amyloid angiopathy, which increases the risk of intracranial hemorrhage [20]. The incidence and severity of intracranial hemorrhage increased with the delay in IVT administration. It may be caused by the hemorrhagic transformation of the coexisting asymptomatic ischemic strokes induced by IVT administration past the safe period [69].

The aforementioned analysis of six studies by Janská et al. [14], which included a study by Chen et al. [20], listed the following complications of standard IVT administered to patients with CRAO within 4.5 h after the onset of symptoms: anaphylactic reaction, laryngeal edema, angioedema, rash, and urticaria [7,14].

The validity of using IVT therapy according to the current stroke protocol within the period of up to 4.5 h as a treatment option for NA-CRAO is supported by the results of the study by Raber et al. It was a retrospective study including 37 patients with NA-CRAO divided into two groups, 21 conservatively treated patients and 16 patients treated with IVT within the 4.5-h period. The mean time from symptom onset to IVT treatment initiation was 158 min. A favorable outcome was obtained in 3 patients (19%) in the IVT group, and in no patients treated with conventional methods. No serious adverse events were observed after IVT [21].

However, it is not certain whether IVT improves the results of NA-CRAO treatment. Recently, the study by Baumgartner et al. provided data from the largest published cohort of RAO patients subjected to IVT to date. After screening 200 patients with acute RAO, 47 patients who received IVT and 34 patients who did not receive IVT were included in the study. In this study, the incidence of bleeding following IVT was low, but it is noteworthy that IVT was not superior to conservative treatment with respect to VA improvement. VA at follow-up was significantly better compared to baseline scores in IVT patients (∆VA 0.5  ±  0.8, *p*  <  0.001) and non-IVT patients (∆VA 0.40  ±  1.1, *p*  <  0.05). During the follow-up period, no significant differences in ∆VA and visual recovery rate were observed between groups [22].

Shahjouei et al. performed a meta-analysis to evaluate the rate of visual recovery after IVT or IAT using tPA or urokinase in patients with NA-CRAO. The study results indicate that early thrombolytic therapy using tPA is associated with more significant VA improvement in patients with NA-CRAO. Visual improvement for ≥0.3 logMAR was reported in 74.3% of patients who received IVT-tPA within 4.5 h (CI: 60.9–86.0%; unadjusted rate: 73.2%) and 60.0% of those who received IAT-tPA within 24 h (CI: 49.1–70.5%; unadjusted rate: 59.6%). A VA of ≥20/100 was observed among 39.0% of patients after IVT-tPA within 4.5 h and 21.9% of those with IAT-tPA within 24 h [23].

The above studies indicate the validity of conducting randomized trials to assess the benefits of IVT in patients with RAO and to determine the optimal time interval for this treatment method.

There are currently three clinical trials underway, the results of which will expand our knowledge on this subject. The studies are A Phase III Randomized, Blind, Double Dummy, Multi-center Study Assessing the Efficacy and Safety of IV Thrombolysis (Alteplase) in Patients With Acute Central Retinal Artery Occlusion (THEIA), ClinicalTrials.gov ID: NCT03 197 194, Early Reperfusion Therapy with Intravenous Alteplase for Recovery of VISION in Acute Central Retinal Artery Occlusion (REVISION), ClinicalTrials.gov ID: NCT04 965 038, and Tenecteplase in Central Retinal Artery Occlusion Study (Ten-CRAOS); ClinicalTrials.gov ID: NCT04 526 951 [25].

Recently, researchers have also considered the combination of thrombolysis and neuroprotection, which may extend the time for initiating effective NA-CRAO treatment and potentially improve the obtained outcomes. Experimental neuroprotection for CRAO includes angiopoietin (Comp Ang1), KUS 121, gene therapy (XIAP—X-linked inhibitor of apoptosis), and hypothermia [70].

#### 3.2.3. Intra-Arterial Thrombolysis

IAT in the treatment of CRAO involves the administration of tPA directly into the ocular circulation via highly selective catheterization of the ophthalmic artery [15]. It may be superior to IVT due to the use of a lower tPA dose, as well as the potential extension of the effective period of drug administration after the onset of symptoms [17]. Thanks to the lower dose reaching the systemic circulation, this method may be considered in patients with systemic contraindications to IVT, such as recent surgery, gastrointestinal bleeding, or coagulation disorders [15]. However, the reduction in systemic complications is accompanied by the risk of arterial dissection, catheter-induced spasm, and atherosclerotic plaque displacement in the ocular circulation with possible distal embolization [15,24]. The dose may vary between 10 and 80 mg, with lower doses administered in boluses instead of continuous infusion. Many studies indicate 60–70% VA improvement in patients subjected to IAT. Its limitation, however, is the fact that its administration requires catheterization of the ophthalmic artery, which can only be performed by highly qualified teams of neurosurgeons [17]. Moreover, IAT is laborious, requires an efficient intervention kit in case of emergency, poses technical challenges (including the risk of spasm induced by the presence of the catheter in the ophthalmic artery), and is associated with the risk of periprocedural stroke [15,24].

The only randomized prospective study comparing the therapeutic efficacy of local intra-arterial fibrinolysis (LIF) using recombinant tissue plasminogen activator (rtPA) to conservative treatment methods in patients with acute NA-CRAO was the European Assessment Group for Lysis in the Eye study (EAGLE) conducted by Schumacher et al. In this study, the patients were divided into two groups. Conservative treatment methods were applied in 40 patients and LIF in 44 patients. Patients in both groups received low-dose weight-adjusted heparin twice daily for 5 days, starting on the first day after the intervention. Next, they were given acetylsalicylic acid at a dose of 100 mg daily for at least 4 weeks. The conservative methods included intravenous heparin, intravenous acetazolamide, eye massage, administration of beta-blockers in drops, hemodilution, and administration of acetylsalicylic acid. The average time to application of treatment in this group was 10.99 ± 5.49 h. The second group of patients received a thrombolytic agent administered into the ophthalmic artery, and, in case of occlusion or severe stenosis of the internal carotid artery, it was administered into the external carotid artery. Thrombolysis was performed using rtPA. Treatment was discontinued after the administration of up to 50 mg of rtPA. After the administration of 15, 30, 45, and 50 mg, a physician assessed the VA score using the Snellen chart and performed a fundoscopic examination to assess changes in the retina. The average time to drug administration in this group of patients was 12.78 ± 5.77 h [18].

The EAGLE study was stopped based on the recommendations of the Data and Safety Monitoring Committee after the first interim analysis because there was no difference in the rate of clinically significant VA improvement between the two groups and a higher rate of adverse events in the LIF group (37.1% vs. 3.4%). The main issue in this study is that no patient was treated with LIF within 4.5 h; only 4 of 41 patients were treated within 6 h (mean time between symptom onset and treatment was 13 h), which reflects how difficult it is to rapidly implement this treatment method [18].

Adverse events that occurred in patients treated with LIF included intracerebral hemorrhage, cerebellar hemorrhage with paralysis, right hemiparesis, disturbances of consciousness, headache, dizziness, and vascular complications such as internal carotid artery spasm, eyelid edema, hematoma at the vessel puncture site, increased IOP, increased troponin levels, tinnitus, oral hemorrhage, post-procedural hemorrhage, epistaxis, and facial hyperaesthesia [18].

#### 3.2.4. RAO as a Stroke Equivalent in the Context of Secondary Prevention

The AHA and ASA define central nervous system (CNS) infarction as brain, spinal cord, or retinal cell death attributable to ischemia [12]. Patients with RAO have a statistically significantly higher risk of stroke, myocardial infarction, and death compared to control groups, with the risk of stroke being the highest in the first weeks after an episode of RAO [11,71,72]. Moreover, Fallico et al. showed that 30% of patients with acute CRAO and 25% of patients with acute BRAO had acute cerebral ischemia on the MRI, both accompanied and not by neurological symptoms. Asymptomatic cerebral ischemia was found in 21% of patients with acute CRAO and 28% of patients with acute BRAO [73].

The association between RAO and subsequent CNS ischemic episodes necessitates prompt referral to the emergency department or stroke unit for neurological evaluation, brain imaging, vascular imaging and cardiac monitoring, and urgent expanded diagnostics to search for the cause of the ischemic incident, as well as evaluation of these patients for concomitant cardiovascular risk factors [13,15,71]. Immediate evaluation results in fast-track access to a specialist, to diagnostic investigation, and to a multidisciplinary network, and allows immediate secondary prevention of cardiovascular disease. The EXPRESS and SOS-TIA studies both demonstrated that prompt evaluation and immediate treatment after transient ischemic attack (TIA) and minor stroke greatly reduced the risk of recurrent stroke (by 80%) [74,75].

The method of secondary prevention of subsequent episodes should be determined by a team of specialists, including a neurologist, ophthalmologist, and internist or primary care physician [15,17]. The neurologist’s role is to determine the cause, initiate an appropriate pharmacological secondary prevention strategy, and work in concert with the patient’s internist/primary care physician to control modifiable risk factors. It is noteworthy that patients with RAO have numerous, often undiagnosed, cardiovascular risk factors. They include hypertension, diabetes, smoking, hypercholesterolemia, and obesity [2]. Furthermore, a recent study by Dropiński et al. showed that patients with CRAO had vascular endothelial damage, thicker intima-media complex thickness, and left ventricular diastolic dysfunction, likely associated with an increased risk of cardiovascular events [76].

Patients with a history of RAO require ongoing ophthalmological care to monitor the healthy eye and monitor for the development of ophthalmological complications in the eye after an RAO episode, which includes the occurrence of neovascularisation and the risk of developing secondary neovascular glaucoma [15].

There are currently no guidelines for antiplatelet therapy for RAO. Nevertheless, given the definition of RAO as stroke equivalent, antiplatelet therapy may be considered in these patients according to the guidelines for TIA or cryptogenic stroke [15,17].

Recommendations for antiplatelet therapy following stroke and TIA are based on the use of the National Institutes of Health Stroke Scale (NIHSS) (Table 2), which assesses the severity of neurological deficit, taking into account the assessment of the state of consciousness, orientation and attention, visual, sensory, motor, and language functions [77], as well as the ABCD2 scale (Table 3), which takes such factors as age (A), blood pressure (B), clinical features (C), duration of symptoms (D), and the presence of diabetes (D) into account. The risk assessment may be extended by using the ABCD2—I scale, which includes brain imaging techniques such as computed tomography angiography (CTA) and diffusion-weighted magnetic resonance imaging (DWI MRI), as well as ultrasound assessment of the degree of large artery atherosclerosis [78]. Recently, Zafar et al. recommended a new scoring system, “ABCD2E”, also emphasizing the need to consider visual symptoms, including sudden transient vision impairment, blurry vision, double vision, or loss of vision [79].

**Table 2:** National Institutes of Health Stroke Scale (NIHSS) [77].

Assessed Element	Assessed Function	Reaction and Result
1A	State of consciousness	0—Alert 1—Not alert, but arousable by minor stimulation 2—Not alert, obtunded 3—Unresponsive
1B	Questions assessing consciousness (2)	0—Both answers right 1—One answer right 2—Both answers wrong
1C	Commands assessing consciousness	0—Performs both tasks correctly 1—Performs one task correctly 2—Fails to perform either task
2	Conjugated gaze	0—Normal 1—Partial gaze palsy 2—Complete gaze palsy
3	Visual field	0—Correct visual field 1—Partial hemianopia 2—Complete hemianopia 3—Bilateral hemianopia
4	Facial palsy	0—Normal 1—Minor paralysis 2—Partial paralysis 3—Complete paralysis
5	Arm motor drift (a) left (b) right	0—No drift 1—Drift within 10 s 2—Falls within 10 s 3—No effort against gravity 4—No movement
6	Leg motor drift (a) left (b) right	0—No drift 1—Drift within 5 seconds 2—Falls within 5 seconds 3—No effort against gravity 4—No movement
7	Limb ataxia	0—No ataxia 1—In one limb 2—In two limbs
8	Sensation	s0—Normal 1—Mild sensory loss 2—Severe sensory loss
9	Language	0—Normal 1—Mild aphasia 2—Severe aphasia 3—Mute, global aphasia
10	Dysarthria	0—Normal 1—Mild dysarthria 2—Severe dysarthria
11	Extinction or inattention	0—Normal 1—Mild (affecting a single sense) 2—Severe (affecting two senses)
Score = 0–42		

The need for appropriate treatment of ischemic stroke and TIA results from the high risk of stroke recurrence [26]. In order to reduce the rate of stroke recurrence, dual antiplatelet therapy has gained increasing attention in recent years. The Clopidogrel in High-Risk Patients with Acute Nondisabling Cerebrovascular Events (CHANCE) study and the Platelet-Oriented Inhibition in New TIA and Minor Ischemic Stroke (POINT) study showed that in patients with minor non-cardioembolic ischemic stroke or high-risk TIA, the addition of clopidogrel to aspirin reduces the risk of stroke recurrence. The Acute Stroke or Transient Ischaemic Attack Treated with Ticagrelor and Acetylsalicylic Acid for Prevention of Stroke and Death (THALES) study evaluated the association of ticagrelor and aspirin in mild-to-moderate non-cardioembolic ischemic stroke or high-risk TIA, showing a reduced risk of subsequent stroke compared to aspirin alone. Recently, the use of dual antiplatelet therapy has been implemented in guidelines [26]. A comparison of guidelines for the use of antiplatelet therapy in patients after ischemic events is presented in Table 4 [26]. According to the literature, BRAO and CRAO are best classified as minor strokes [13]. In case of non-cardioembolic minor ischemic stroke (NIHSS score of 3 or less) or high-risk TIA (ABCD2 score of 4 or more), the European Stroke Association (ESO) and the American Heart Association/American Stroke Association (AHA/ASA) recommend the use of DAPT, followed by single antiplatelet therapy (Table 4) [26].

Currently, there are no clear recommendations regarding the indications for anticoagulation treatment after an episode of RAO. At this point, it is worth mentioning AF, which is one of the most common arrhythmias diagnosed in patients with RAO. Due to the fact that the risk of stroke in patients with AF and RAO is higher compared to AF patients who have not had RAO, it is reasonable to extend the diagnostics after an episode of RAO to include AF. Currently, RAO is considered the equivalent of a stroke, and in this context, a patient with AF after an episode of RAO should receive 2 points on the CHA2DS2-VASc scale (Table 5), which means that he is at high risk of thromboembolic complications, implying the need to introduce appropriate anticoagulation treatment [80]. Appropriate treatment can reduce the incidence of stroke in AF patients by up to 80% [81].

The effectiveness of antiplatelet and/or anticoagulation therapy in preventing subsequent ischemic events, including stroke, myocardial infarction, and death in patients with RAO, has been assessed in a few studies [72,82,83]. In the study by Hankey et al., treatment with aspirin and anticoagulation did not have a significant effect on the risk of subsequent stroke [82]. A study by Kang et al. also failed to demonstrate the benefit of aspirin in reducing the risk of stroke after RAO [83]. In the study by Vestergaard et al., treatment with aspirin did not change the risk of stroke, myocardial infarction, or death in the first year after RAO, however after one year, it was associated with a reduction in the risk of both strokes (adjusted RR, 0.80; 95% CI, 0.68–0.94; *p* = 0.0058) and death (adjusted RR, 0.86; 95% CI, 0.79–0.93; *p* < 0.001). Clopidogrel was associated with a reduced risk of death after one year, with an adjusted RR of 0.84 (95% CI, 0.74–0.95; *p* = 0.0068). Anti-coagulant treatment was associated with a reduction in the risk of stroke at all time intervals and of myocardial infarction and death at 90 and 365 days, respectively [72].

Recent reports have also indicated that the use of statins after ischemic stroke reduces the risk of recurrent stroke; in the case of RAO, this issue needs to be investigated in more detail. However, the results of the study by Yoo et al. indicate that patients newly diagnosed with RAO receiving treatment with statins, especially in the long term, were associated with a low risk of future cardiovascular events [27,28].

### 3.3. Nd:YAG Laser Embolysis or Embolectomy

The methods of RAO treatment could also include the use of transluminal Nd/YAG laser embolysis (TYL) for mechanical fragmentation of the embolus. The procedure is used to treat both CRAO and BRAO in cases when the embolic material is visible [53,54,55,56,57,58,59]. In cases of transluminal Nd:YAG laser embolectomy (TYE), the laser is focused on the occluded vessel, the vessel wall is ruptured, and the embolism dislocates to the vitreous humor [58].

The technique should be initiated with the lowest possible pulse energy and gradually increased until the embolus has been fragmented. The fragments migrate to the peripheral parts of the vessel with the blood flow. Studies indicate the possibility of using combined thrombolytic therapy or HBO therapy in order to increase the effectiveness of treatment [54].

The most frequently observed complications include preretinal hemorrhage and bleeding into the vitreous humor, which in some cases contributes to the need for vitrectomy. Other possible complications include choroidal neovascularisation and epiretinal membrane formation [53].

In a systematic review and meta-analysis, Man et al. analyzed data from clinical trials and case reports regarding the use of transluminal Nd:YAG laser embolectomy/embolysis for CRAO or BRAO in 61 patients. The key observation was a significant VA improvement after the procedure in 87% of the analyzed clinical cases. It was concluded that the use of higher pulse energy (≥2.4 mJ) did not improve treatment outcomes but was associated with a high percentage (54%) of post-procedural complications in the form of vitreoretinal hemorrhage, which led to the need for vitrectomy in 18% of cases. The authors of the publication were not able to clearly answer the question of whether the potential benefits of using this method of treatment justified the risk associated with the procedure, but further research was considered reasonable [55].

Mehboob et al. conducted a randomized clinical trial in 14 patients. All the participants were treated within 24 h after the onset of RAO symptoms. The patients were randomly divided into two groups. Both groups received pretreatment with ocular massage, antiglaucoma therapy, sublingual administration of glyceryl trinitrate, and anterior chamber paracentesis to reduce the IOP. Moreover, a group of 7 patients was subjected to TYE. In the group of patients who were not treated with TYE, reperfusion was achieved in two patients (28.6%), and a significant improvement in VA to over 6/60 was achieved in 3 patients (42.8%). In the TYE group, reperfusion was observed in 5 patients (71.4%), while significant VA improvement was achieved in 6 patients (85.7%). In all eyes that showed functional improvement, Nd:YAG laser embolysis was performed within a period of up to 6 h after the onset of symptoms. On the basis of the obtained results, the authors concluded that TYL is more effective in the management of fovea threatening RAO compared to conventional treatment methods if the procedure is performed within a period of up to 6 h after the onset of symptoms. The small number of study subjects constituted a study limitation. Furthermore, post-procedural complications were not assessed in this study [56].

Opremcak et al. presented the results obtained in a non-randomized study of 19 patients who underwent Nd:YAG embolysis or embolectomy. Fundoscopic examination and fundus fluorescein angiography (FFA) confirmed retinal reperfusion. VA improvement was achieved in 89% of patients. An important aspect was the high rate of intraoperative complications, which affected 47% of patients. Seven subjects experienced vitreous hemorrhage, and one patient experienced subhyaloid hemorrhage. Moreover, vitrectomy was necessary in 5 patients [57].

Much et al. performed a retrospective analysis of data from 5 patients subjected to TYE. The time of the procedure varied from 4 to 30 h after the onset of RAO symptoms. VA improvement was achieved in 2 patients. All procedures resulted in vitreous hemorrhage, but only one patient required surgical intervention [58].

Chai et al. performed a retrospective analysis of 34 cases in which TYL was combined with IVT using urokinase. Imaging of the fundus and FFA were performed before and immediately after the TYL procedure. In all patients, IVT using urokinase at a dose of 10–20 u/d was applied on the day after the procedure and continued for 5 days. Following TYL, FFA revealed that the CRA and its branches showed complete restoration of blood flow in 13 patients (38.2%) and major restoration in 11 patients (32.4%). Following the subsequent IVT using urokinase, FFA demonstrated that the CRA and its branches showed complete restoration of blood flow in 16 cases (47.1%) and major restoration in 15 cases (44.1%). During laser treatment, retinal hemorrhage occurred in 2 patients and vitreous hemorrhage in 1 patient. No vitreous hemorrhage or systemic complications were observed in the course of IVT treatment. No cases of retinal or choroidal neovascularisation were observed during the follow-up period, which lasted for 6 to 14 months [59].

The results of studies on the use of the Nd:YAG laser in RAO are promising, but this method still requires reliable evaluation.

### 3.4. Vitrectomy

The use of pars plana vitrectomy (PPV) in the treatment of RAO involves the manipulation of intraocular and arterial pressures. The change in local pressure is intended to increase the mobility of the embolus and move its fragments to the distal parts of the vessel, thereby reducing the extent of ischemia. Vitrectomy techniques used to treat RAO also include retinal artery massage, CRA cannulation with microneedle, bloodletting, embolectomy, and vitrectomy with arteriotomy and neurotomy [60,61,62,63,64,65].

Okonkwo et al. described a case of a patient with CRAO in whom the pressure difference was induced during surgery by lowering the IOP and simultaneously increasing the blood pressure in order to increase the ability of the embolus to move. This method can be used to achieve effective reperfusion along the entire CRA [60].

Embolus displacement alone does not fully solve the problem of ischemia, so this method has been modified to include its surgical removal.

Garcia-Arumi et al. used the method of surgical removal of the embolus by making a longitudinal incision of the anterior wall of the occluded arteriole using a microvitreoretinal blade. This method enabled reperfusion of the occluded artery and VA and visual field improvement in 71% of patients [61]. However, Cisiecki et al. indicated that the use of vitrectomy with arteriotomy or with neurotomy and arteriotomy in patients in whom conservative treatment was ineffective did not provide any benefit in terms of improving VA after the procedure [62]. It is worth noting that Cisiecki et al. presented a case series consisting of 11 cases of CRAO and 1 case of BRAO [62], while the work of Garcia-Arumi et al. concerned mainly cases of patients with BRAO and only 1 case of CRAO, in whom the treatment was unsuccessful [61].

Furthermore, the study by Garcia-Arumi et al. included patients with RAO symptoms lasting < 36 h, with 5 of 7 patients achieving VA improvement. A particularly significant improvement in VA was observed in a female patient, in whom intervention was initiated 22 h after the onset of symptoms. In this patient, VA improved from counting fingers to 20/30. The significant improvement of VA in patients who received treatment such a long time after the onset of symptoms distinguishes this work from others included in this review and encourages verification of the results presented therein [61].

The presented CRAO treatment method, despite its potential effectiveness demonstrated mainly on the basis of the analysis of a series of clinical cases, is burdened with potential complications in the form of vitreous hemorrhage [61]. Further studies are needed to confirm its usefulness in the treatment of CRAO.

### 3.5. KUS121

Studies by Ikeda et al. on the use of KUS121 in the treatment of RAO seem promising. A new neuroprotective agent, KUS121, is an ATPase inhibitor of valosin-containing protein (VCP). The results of the first-in-human Phase 1/2 clinical trial indicate that KUS121, administered as an intravitreal injection to patients with CRAO, is both safe and effective in improving visual function. According to the study authors, KUS121 clearly inhibits the decrease in the intracellular adenosine triphosphate (ATP) concentration and, consequently, inhibits the endoplasmic reticulum stress and cell death, in this case, the inner retinal neurons and the functions of the surviving neurons are restored by better retinal blood flow or by diffusion of choroidal blood flow [66].

The A Study of the Efficacy and Safety of KUS121 in Participants With Acute Non-Arthritic Central Retinal Artery Occlusion (CRAO) (GION); ClinicalTrials.gov ID: NCT06178055, double-masked, sham-controlled, multi-center, parallel-group, phase II study has been initiated in order to further assess the efficacy and safety of KUS121 in the treatment of CRAO.

## 4. Conclusions

The lack of clear guidelines regarding the most effective way to treat RAO constitutes a significant problem. There are concerns regarding the lack of clear evidence on the effectiveness of individual methods and the high-risk profile of those proven to be more effective. Large, multi-center, randomized controlled trials are needed to evaluate current treatment options and to develop clear guidelines for the treatment of RAO. Moreover, determining the optimal time frame for initiating therapy is, therefore, one of the key issues for future research.

The most promising RAO treatment method is IVT, which is a standardized method for the treatment of ischemic stroke. Ongoing studies on the use of different fibrinolytic agents will provide greater insight into thrombolytic therapy in RAO cases.

Due to the proven high risk of stroke recurrence after RAO, secondary prevention against another ischemic event plays a fundamental role. Recommendations for secondary prevention must be based on interdisciplinary cooperation. Due to RAO being considered a minor stroke, dual antiplatelet therapy followed by antiplatelet therapy is recommended. Recognition of RAO as a stroke requires awarding 2 points on the CHA2DS2-VASc scale for patients after RAO with atrial fibrillation, which is crucial for initiating anticoagulation treatment.

In our opinion, social campaigns are necessary to emphasize that a sudden, painless loss of vision in one eye may indicate a stroke, and the time to make a diagnosis and start treatment is crucial for the prognosis.

## Figures and Tables

**Table 1:** Characteristics of treatment methods used in RAO.

Method	Mechanism	Best Time for the Implementation of Treatment After the Onset of Symptoms	Efficacy	Complications	Limitations
PXF [7,33,34,47]	Decreased red blood cell rigidity, reduced blood viscosity, reduced potential for thrombus formation	No data	VA deterioration despite therapy, increased PSF and EDF	Dehydration, constipation, anorexia, cholecystitis, aseptic meningitis, seizures, confusion, depressive symptoms, hypotension, edema, dyspnea, epistaxis, respiratory distress, dermatitis, angioedema, urticaria, pruritus, otalgia, scotomas, conjunctivitis, and blurred vision	Allergy to theophylline or caffeine
Rebreathing into a bag [17,35,36]	Vasodilation	No data	Induction of hypercapnia and hypoxia—increase in ocular perfusion pressure—displacement of embolus	Systemic risk and the risk of more severe retinal hypoxia, blood pressure lowering	Only for young people with no history of cardiovascular disease
Carbogen [17,36,37,38]		Within 24 h after the onset of symptoms [37]	Unconfirmed; no change in arterial diameter and a significant increase in the vascular flow rate and arteriovenous passage time were observed	Blood pressure lowering	No data
Nitroglycerine [7,17,39]		No data	Cannot be established; only used in combined treatment	Blood pressure lowering	Severe anemia, recent use of phosphodiesterase inhibitors
HBOT [6,40,41,42,43,44,45,46]	Increased oxygen concentration in the blood	24 h	May meet 100% of the retina’s demand for oxygen	Barotrauma, ear pain, ruptured eardrum, and generalized seizures	No data
Eye massage [18,39,46,47,48]	IOP reduction leading to retinal perfusion pressure increase	No data	No studies available; used in combination with other methods; lacks significant efficacy	No data	No data
Acetazolamide [18,39,47,48,49]		No data	Increased blood flow in the retina	Paraesthesia, metallic/bitter taste in the mouth, fatigue, anaphylaxis, metabolic acidosis, Stevens–Johnson syndrome, and blood dyscrasia	No data
Anterior chamber paracentesis [17,37,46,47,48,50]		Within 24 h after the onset of symptoms [36]	No significant VA improvement was observed	Trauma to intraocular structures, corneal decompensation due to the iridocorneal touch, intraocular hypotony, hemorrhage, and intraocular infection	It may potentially work only in cases of embolic CRAO and not arterial occlusion caused by thrombi, vasospasm, arteritis, or dissecting aneurysm
Methylprednisolone [51,52]	Decrease in vascular endothelial edema	Not determined	Data based on a study of a series of 4 clinical cases; high efficacy in cases of vasospasm or CRAO secondary to vasculitis	Rare, but may be serious, including anaphylactic reactions, tetraplegia, cardiac arrhythmia, and sudden death	Lack of efficacy in RAO other than caused by vasculitis
IVT [7,14,15,16,19,20,21,22,23,25]	Dissolving the clot causing CRA stenosis with an intravenous thrombolytic agent	Within 4.5 h after the onset of symptoms	The percentage of patients with significant VA improvement varies in respective studies, but it is generally in the 40–50% range	Intracerebral and systemic hemorrhage Anaphylactic reaction, laryngeal edema, angioedema, rash, urticaria	Ophthalmic contraindications: elevated IOP > 30 mmHg, VA > 0.1, temporal arteritis, combined arteriovenous occlusion, CRA branch occlusion, CRAO without foveal ischemia, choroidal neovascularisation, proliferative diabetic retinopathy, or other severe retinopathy. Other contraindications: intracranial hemorrhage or other significant acute or recent bleeding, uncontrolled hypertension, anticoagulation therapy, hemorrhagic diathesis, aneurysm or arterial dissection, infective endocarditis, peripartum period
IAT [15,17,18,24]	Dissolving the clot causing CRA stenosis with an intravenous thrombolytic agent administered directly to the ocular circulation	12.78 ± 5.77 h	VA improvement in as many as 60–70% of patients	Intracerebral hemorrhage, cerebellar hemorrhage with paralysis, right hemiparesis, disturbances of consciousness, headache, dizziness, internal carotid artery spasm, eyelid edema, corneal erosion, hematoma at the vessel puncture site, increased IOP, increased troponin levels, tinnitus, oral hemorrhage, post-procedural hemorrhage, epistaxis, and facial hyperaesthesia	Availability of qualified personnel, time for the implementation of treatment limited to 6 h
TYL/TYE [53,54,55,56,57,58,59]	Extravascular laser application to obtain embolysis or embolectomy	6 h	Significant VA improvement in most cases	Vitreous hemorrhage, need for vitrectomy	Location and visibility of the embolus
Vitrectomy [60,61,62,63,64,65]	Manipulation with ocular perfusion pressure, retinal artery massage, CRA cannulation with microneedle, bloodletting, embolectomy, and vitrectomy with arteriotomy and neurotomy	No clinical data; animal model showed minimal retinal cellular damage 2 h after successful reperfusion	No validated randomized clinical trials; case studies demonstrated the potential effectiveness of this method	Hemorrhage to vitreous cavity	The location and structure of the embolus may affect the outcome of the procedure. The presence of infectious disease within the eye adnexa, poor general condition, allergy to anesthetics
KUS121 [66]	Inhibits the decrease in intracellular ATP concentration	Ongoing studies	Ongoing studies	Adverse events not defined as complications: IOP increase, subconjunctival hemorrhage and corneal epithelial damage, neovascularisation of the iris	Ongoing studies

CRA, central retinal artery; CRAO, central retinal artery occlusion; EDF, end-diastolic flow; HBOT, hyperbaric oxygen therapy; IAT, intra-arterial thrombolysis; IVT, intravenous thrombolysis; KUS, Kyoto University Substance; PSF, peak-systolic flow; PXF, pentoxifylline; TYE, transluminal Nd:YAG laser embolectomy; TYL, transluminal Nd/YAG laser embolysis; VA, visual acuity.

**Table 3:** Components of the ABCD2 score [78].

Abbreviation (Mnemonic: ABCD2)	Parameters	Score
A	Age (years) >60	1
B	Blood pressure (mmHg) SBP > 140 or DBP > 90	1
C	Clinical features: Unilateral weakness Speech disturbance without weakness	2 1
D	Duration of symptoms (minutes): ≥60 <10–59 min	2 1
D_2_	Diabetes	1

DBP, diastolic blood pressure; SBP, systolic blood pressure.

**Table 4:** The European Stroke Association and the American Heart Association/American Stroke Association recommendation for dual antiplatelet therapy [26].

Guidelines		DAPT in Non-Cardioembolic Ischemic Stroke and High-Risk TIA		DAPT in Stroke and TIA Due to Intracranial Stenosis
AHA/ASA		aspirin + clopidogrel	aspirin + ticagrelor	aspirin + clopidogrel
	Patients	minor ischemic stroke (NIHSS ≤ 3)	minor to moderate stroke (NIHSS ≤ 5)	stroke or TIA attributable to severe stenosis (70–99%) of a major intracranial artery
		high-risk TIA (ABCD2 ≥ 4)	high-risk TIA (ABCD2 ≥ 6 or symptomatic intracranial or extracranial ≥ 30% stenosis of an artery that could account for the event)	
	interval between the event and the introduction of therapy	ideally 12–24 h, at least 7 days	24 h	30 days
	duration of DAPT	21–90 days	30 days	90 days
	strength of recommendation	strong	weak	moderate
ESO		aspirin + clopidogrel	aspirin + ticagrelor	aspirin + cilostazol or clopidogrel or ticagrelor
	Patients	minor ischemic stroke (NIHSS ≤ 3)	mild to moderate ischemic stroke (NIHSS ≤ 5)	ischemic stroke or TIA related to intracranial stenosis due to ICAD
		high-risk TIA (ABCD2 ≥ 4)	high-risk TIA (ABCD2 ≥ 6) or intracranial atherosclerotic disease or at least 50% stenosis in an internal carotid artery that could account for the presentation	
	interval between the event and the introduction of therapy	24 h	24 h	no specific indication
	duration of DAPT	21 days	30 days	90 days
	strength of recommendation	strong	weak	weak

ABCD2: (age, blood pressure, clinical features, duration of symptoms, and diabetes) score; AHA/ASA, American Heart Association/American Stroke Association; DAPT, double antiplatelet therapy; ICAD, intracranial atherosclerotic disease; NIHSS, National Institutes of Health Stroke Scale; TIA, transient ischemic attack.

**Table 5:** Components of the CHA2DS2-VASc scale [80].

	Risk Factor	Description	Score
C	Congestive HF, clinical HF, moderate or severe LV dysfunction or HCM	Recent decompensated HF, irrespective of LVEF (HFrEF or HFpEF) or presence of moderate or severe LV systolic function impairment (also asymptomatic) in cardiac imaging	1
H	Hypertension	Resting blood pressure > 140/90 mm Hg ≥ 2 measurements taken on different occasions or appropriate hypotensive treatment	1
A_2_	Age >75 years	-	2
D	Diabetes	(1) Random venous blood glucose ≥ 200 mg/dL (≥11.1 mmol/L) + symptoms of diabetes (2) Double measurement of fasting blood glucose ≥ 126 mg/dL (≥7 mmol/L) (3) OGTT ≥ 200 mg/dL (≥11.1 mmol/L)	1
S_2_	History of stroke/TIA/ /thromboembolic event	History of stroke, TIA or peripheral embolism (including RAO)	2
V	Vascular disease	History of myocardial infarction, atherosclerotic peripheral artery disease, atherosclerotic plaque in the aorta	1
A	Age 65–74 years	-	1
Sc	Female gender	Increases the risk if ≥ 1 other risk factor is present	1

HCM, hypertrophic cardiomyopathy; HF, heart failure; HFrEF, heart failure with reduced ejection fraction; HFpEF, heart failure with preserved ejection fraction; LV, left ventricle; LVEF, left ventricular ejection fraction; OGTT, oral glucose tolerance test; RAO, retinal arterial occlusion; TIA, transient ischemic attack.

## Data Availability

Not applicable.
